# Annotations

**Published:** 1908-10-03

**Authors:** 


### Annotations.

### The Tuberculosis Congress.

The opening ceremonies in connection with the great International Congress on Tuberculosis were held on Monday, September 28, at Washington, in the new National Museum. Mr. Cortelyou presided as the personal representative of President Roosevelt. He extended a cordial welcome to those present at the meeting, which was attended by government officials, public-health authorities, and scientists from all over the world. In replying for Great Britain Dr. Newsholme stated on behalf of Mr. Burns that the Local Government Board is shortly about to issue an order compelling Poor-law medical officers to notify any sanitary authorities making application for such a notification, of all cases of phthisis occurring amongst parochial patients. This order, he said, will also render it obligatory on other Poor-law officials to notify changes of address of parochial patients suffering from phthisis. Dr. Newsholme drew attention to the success which has attended recent sanitary work in Great Britain in suppressing tuberculosis, even when not primarily undertaken for that end. The gradual improvement which has taken place in social conditions, such as feeding, housing, and clothing has already begun to bear fruit in this direction; and the modern tendency towards institutional rather than domestic treatment of disease has distinctly reduced the risks of infection from tuberculosis. In this connection Dr. Newsholme warmly praised the work of Lady Aberdeen in Ireland. The Congress is divided into seven sections, at which a number of important papers are being read, while the progress in the war against tuberculosis throughout the world is illustrated by a tuberculosis exhibition held in the same building.

### Tempora Mutantur.

This year as many as four of the London medical schools are dispensing with the old established custom of the "opening address"; they are, St. Bartholomew's, Guy's, St Thomas's, and Westminster. Old customs proverbially die hard, but it really seems as if this particular one is now on its last legs; and, on the whole, perhaps it is better so. The opening address was the product of a more leisurely age than ours, one in which even a somewhat dilettant study of medicine and surgery enabled the youth of average common sense to qualify himself for admission to the Medical Register in four years. An age in which the only semi-medical science not in its infancy was anatomy, and in which anatomy constituted the major part of surgery. Not everything is changed or changing, and so many intricate sciences claim the attention of the medical student that even the first day of the session can hardly be spared for the function which, however much it may add an air of scholarly distinction to the school, is of no sort of use as far as examinations go. Much as we may regret the tendencies of the day, the hustling spirit of prosaic utility which is making short work of an ancient and honourable institution, we cannot shut our eyes to the fact that impatience to get to work is a healthy sign. After all, the opening address, unless the work of one of those rare beings in whom are combined the scholar, the wit, and the man of the world, is apt to degenerate into a string of amiable platitudes not untouched by pedantry. The modern youth needs little reminding that diligence is required of him; the array of subjects waiting to be mastered is quite sufficient warning of that, and he generally avoids as the pestilence the address which is supposed to be composed especially for his benefit, unless the orator is exceptionally popular. Distinguished as are the various gentlemen who undertook this *rôle* at the various schools on October 1, and admirably as they may have sustained its best traditions, it is permissible to wonder whether the time and trouble they spent were productive of adequate result, and how long it will be before the example of the four schools we have mentioned is followed throughout the medical firmament.

### Factitious Athletic Feats.

At the recent annual meeting of the British Medical Association Dr. Leonard Hill, F.R.S., recorded some experiments which demonstrated pretty clearly that if an athlete inhaled oxygen before running he was likely to improve his record, at any rate over courses up to half a mile. The important bearing of these observations on the physiology of respiration was duly pointed out. It is hard, however, to take seriously the suggestion—given in some detail—that in future oxygen cylinders should form part of the equipment of the running-track. Even from the sportsman's point of view there can be no interest in the performances of artificially stimulated competitors; and, indeed, at the Olympic Games just past the use of drugs of any sort was expressly forbidden. It may be remembered, too, that a year or two ago the Jockey Club took pains to stamp out a practice of "doping" racehorses, which had been introduced from America. Surely it is also very probable that fatigue and breathlessness following in the ordinary way upon strong muscular activity act as a natural safety-check, and that some of the dangerous results of over-exertion may follow if they are artificially prevented. These reflections are seasonable, because oxygen-inhalation in athletics has lately come under general notice in connection with an attempt to swim the Channel. No lasting restorative effect seems to have been gained; and indeed the patient is said to have remarked afterwards that he thought the final result the reverse of helpful. This is as it may be, and no doubt Drs. Hill and Flack take a keen interest in further verification of the undoubtedly interesting and striking results above referred to. We may be permitted, however, to express the hope that the athlete of the future will not try to obtain stamina from a gas-bag. Already far too many people waste time striving against natural inaptitude for various pastimes. The born athlete needs about as little stimulation as does the national taste for athletics.

